# Bottlenecks in biobased approaches to plastic degradation

**DOI:** 10.1038/s41467-024-49146-8

**Published:** 2024-06-03

**Authors:** Amelia R. Bergeson, Ashli J. Silvera, Hal S. Alper

**Affiliations:** 1 McKetta Department of Chemical Engineering, The University of Texas at Austin, Austin, TX USA; 2 Institute for Cellular and Molecular Biology, The University of Texas at Austin, Austin, TX USA

**Keywords:** Materials science, Environmental biotechnology, Industrial microbiology, Bioremediation

## Abstract

Plastic waste is an environmental challenge, but also presents a biotechnological opportunity as a unique carbon substrate. With modern biotechnological tools, it is possible to enable both recycling and upcycling. To realize a plastics bioeconomy, significant intrinsic barriers must be overcome using a combination of enzyme, strain, and process engineering. This article highlights advances, challenges, and opportunities for a variety of common plastics.

## Abstract (web summary)

The way we generate and treat plastic waste is a key consideration in the transition from a take-make-waste model to a truly circular economy. Here, authors discuss their views on how biotechnology could contribute to solving the plastic problem, and the barriers we need to overcome to make such approaches a reality.

## Introduction

Plastic has become an ever-present and ubiquitous fixture in our daily lives. Due to their varied characteristics, plastics have a wide range of applications from medical implants to food packaging. Owing to a low cost and rapid production, plastics quickly became single-use items and these same features perpetuate their overuse. Since the commercial introduction of plastics in the 1950s, yearly plastic production has increased over one hundred-fold with cumulative plastic production estimated to be in the tens of thousands of million metric tons by 2050^1^. At current estimates, nearly 400 million tons of plastic will be produced this year—a number equivalent to the collective mass of every human on the planet. Across all this plastic, only about 14% is ultimately recycled^2^. Unrecovered plastics that end in landfills or oceans can persist, leading to an accumulation of plastic in the natural environment and ultimately causing severe environmental consequences^3^. As an alternative, this plastic waste can serve as a potent feedstock for both bio-enabled recycling and upcycling.

## Challenges with plastic end-of-life: a role for a bio-enabled circular economy

**Fig. 1:**
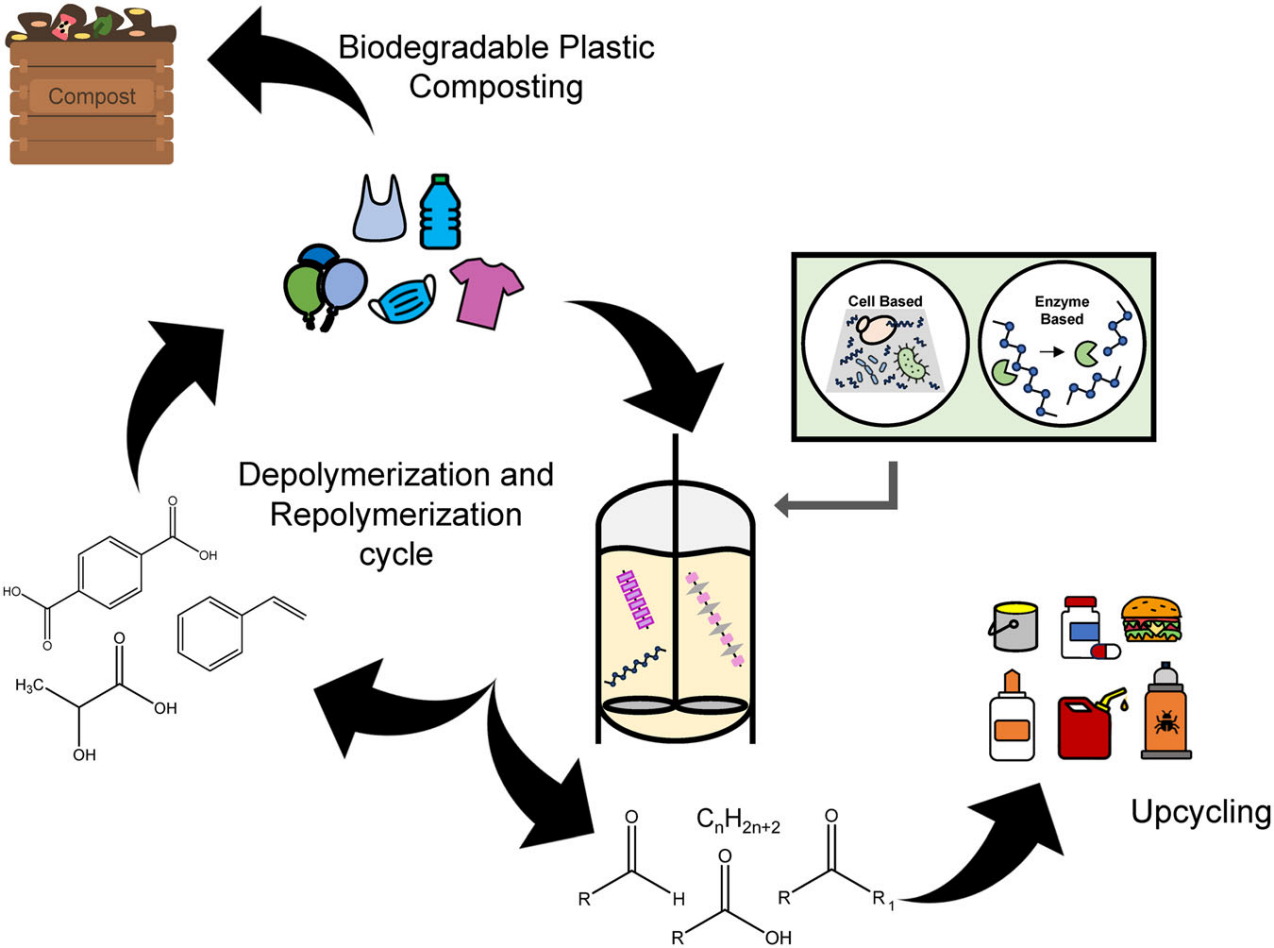
Life cycle of plastic waste. Possible pathways for plastic waste utilization and repurposing include bio-enabled depolymerization/repolymerization, composting, and upcycling. Depending on the approach used (cell-based or enzyme-based) as well as plastic type, biodegradation may result in either the generation of directly-reusable plastic monomers or oligomers. The biodegradation product generated will then dictate the next phase of the waste plastic’s life cycle and hence entry-point into the biorefinery cycle. Original monomers are more convenient starting points for repolymerization whereas the production of oligomers is more conducive to upcycling.

The mechanical and chemical characteristics that make plastics functional and attractive as materials are also the same traits that make biodegradation challenging. The deployment of plastics in the built and natural environment for applications such as underground storage tanks that can persist for decades highlight the extreme recalcitrance of these materials to both chemical- and bio-degradation. However, a full circular bioeconomy involves depolymerization of these materials back into either original monomers (to enable a full polymer recircularization) or constituent compounds (such as oligomers or modified backbones) that can be upcycled into new products in a biorefinery scheme (Fig. 1). As such, at a minimum, effective degradation methods are necessary for the most highly produced, non-biodegradable plastic species such as: polyethylene (PE), polypropylene (PP), polyvinyl chloride (PVC), polyethylene terephthalate (PET), polyurethane (PUR), and polystyrene (PS)^4^.

Nonbiological routes do exist to depolymerize these most abundant polymer species. For example, conventional decomposition of PE is achievable via photodegradation, thermal degradation, and chemical catalytic degradation. While these technologies can produce a mixture of oils and fuels from PE, they also suffer from drawbacks such as a reliance on chromophores for degradation, high energy demands, as well as the production of byproducts which can be toxic to human health^5–7^. Pyrolysis, for example, typically requires temperatures up to 500 °C to degrade some plastics^8^. Additionally, while pyrolysis has the advantage of processing multiple plastic input streams simultaneously, it ultimately results in homogenized products such as pyrolysis oil, gas, and char^8^. Unlike these high-temperature processes, biodegradation can take place at temperatures an order of magnitude lower^9^. Moreover, unlike the homogenization that occurs with processes like pyrolysis, biodegradation can stepwise extract individual products from each plastic species owing to reaction specificity of the enzyme or microbes being used. The result is a true “biorefinery” for plastic wherein plastics may be specifically broken down into constituents and then refactored into the original plastic, new plastics, or new products. In essence, microbial and/or enzyme degradation of mixed-waste plastics has the potential to maximally extract the chemical potential of polymers by creating specific product streams (rather than a homogenized product) that can be recycled and upcycled for a variety of downstream applications (Fig. 1). This feature is especially desirable for plastics which cannot be recycled into a non-homogenized product (such as layered plastics, textiles^10^, and low-quality plastic products) that would otherwise be combined into a mixed pyrolysis oil via conventional approaches. In a biorefinery scheme, each of the depolymerized components can be individually and optimally converted into their own product of interest while maximizing carbon yield and redox potential. This approach is the equivalent of specifically and independently converting the cellulose, hemicellulose, and lignin streams of lignocellulosic biomass into products rather than using a hydrothermal liquefaction product of the total biomass.

**Fig. 2:**
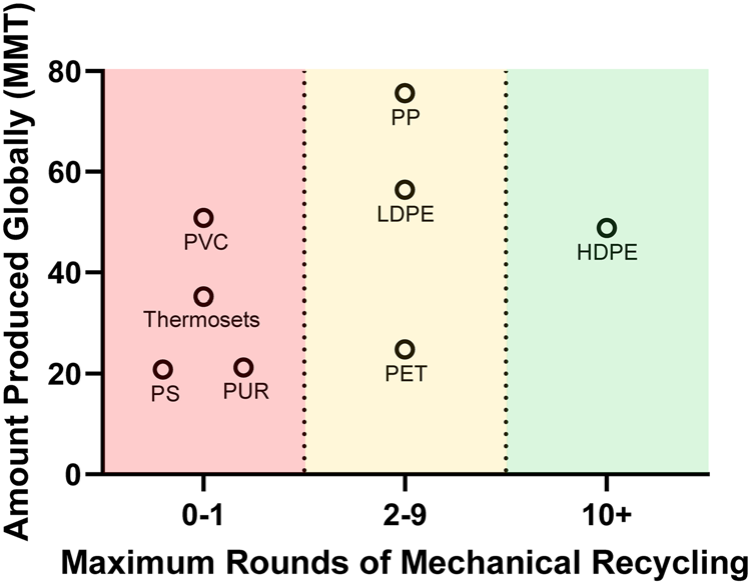
Plastic production by type and mechanical recyclability. Plastic is not infinitely recyclable through traditional means. The temperature and processing during mechanical recycling can result in degraded polymer and material properties which limits the number of times a plastic can be mechanically recycled^82–86^. The number of times a plastic can be mechanically recycled is also drastically impacted by the initial feedstock, the addition of chemical additives, processing temperature, and blending different polymer types together. When these traits are paired together with overall plastic production data (adapted from ref. ^87^), it is clear that new technologies are required to improve the reuse-capability of most of the highly-produced plastics.

Biodegradation is only one component in a circular economy for plastics along with technologies such as chemical and mechanical recycling. The chemical structure of certain polymers (PET as one good example) enables facile scission back into original monomers, thus enabling a theoretically infinitely circular process. At the same time, this polymer is also amenable to several rounds of mechanical recycling before property traits begin to decay and thus mechanical recycling combined with biodegradation and repolymerization can in theory maximally optimize polymer reuse while minimizing total, overall cost^11–13^. It is thus important to balance mechanical and enzymatic recycling blends properly to minimize overall costs and energy input. However, not all polymers are the same with respect to either scale of production or suitability for multiple rounds of mechanical recycling (Fig. 2). The vast majority of plastics (with HDPE as an outlier) can only be minimally recycled via mechanical techniques before polymer integrity is compromised. As a result, it is necessary to develop new technologies to enhance net recyclability (either mechanically, chemically, or biologically) for most of the polymers of interest. The most desirable trait would be the combined trait of high demand coupled with high recyclability (i.e., products that would fall within the upper right quadrant of Fig. 2).

**Fig. 3:**
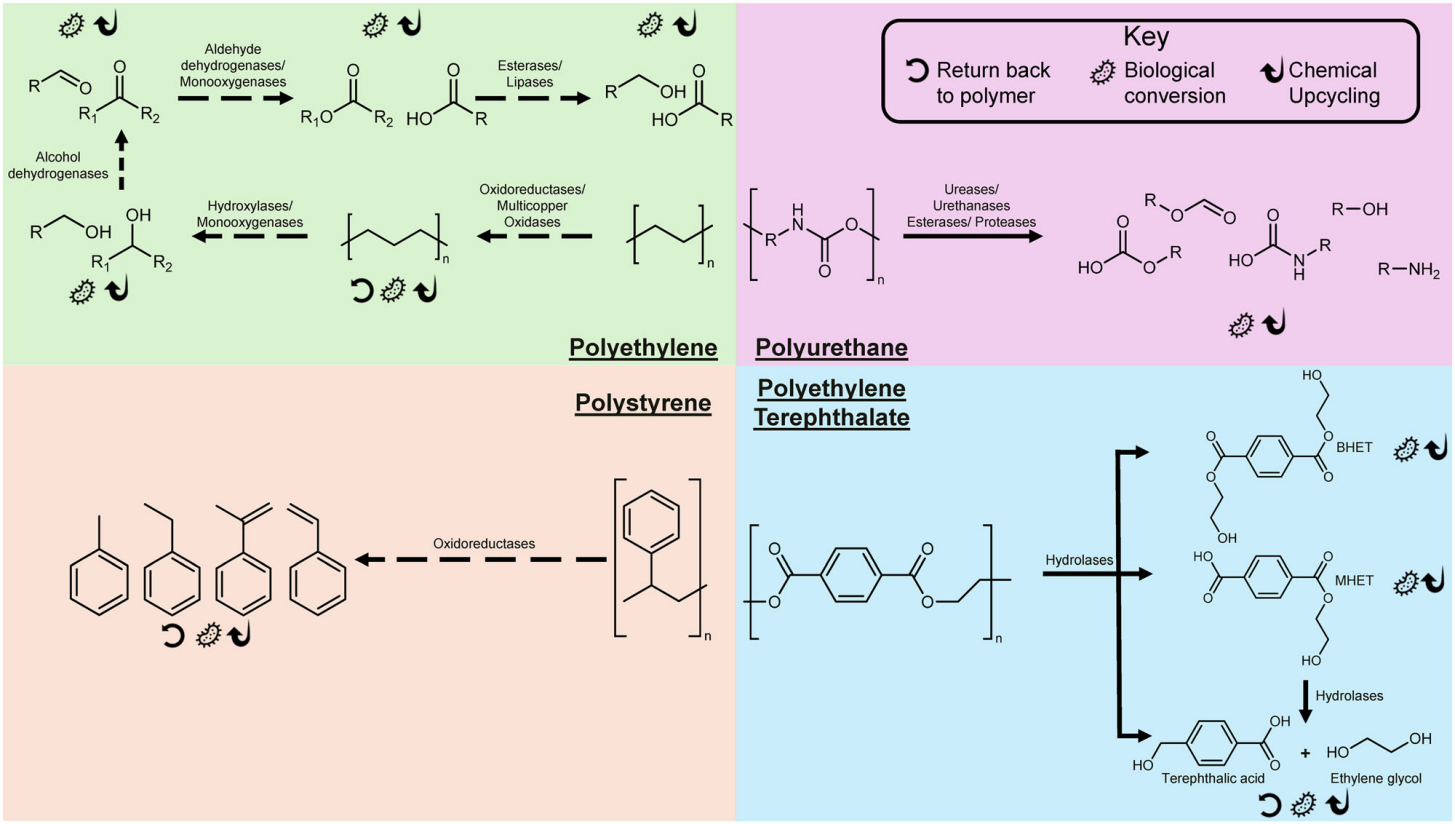
Proposed enzymatic pathways for conversion of multiple plastic types. Enzymatic conversion of plastic waste presents opportunities for coupling other treatments, both chemical and further biological, to produce a wide range of products. In some instances, the constituent monomers generated from enzymatic degradation present opportunities for conversion into new product while others pathways can yield the original monomer of interest, thus enabling a full end-to-end infinite biorecycling approach. Hypothesized (dashed arrows) and realized pathways (solid arrows) discussed throughout this article are depicted in the figure along with representative small degradation products. The capability to reuse, upcycle and repurpose these molecules is highlighted through the graphical legend.

While efforts have demonstrated the ability to fully depolymerize PET back to original monomers and repolymerize them back into virgin plastic, other polymer chemical structures are not amenable to such a cycle. Plastics such as PE and other polyolefins are better suited as feedstocks for upcycling after biological degradation^14–18^. Furthermore, the typical reaction mechanisms for degrading these more recalcitrant plastics introduce heteroatoms such as oxygen to activate bond cleavage. In doing so, these additions do not result in original monomer generation, but rather result in new feedstock compounds. Collectively, the mechanisms of plastic biodegradation can give rise to fully reusable monomers as well as constituents suitable for downstream biological or chemical upcycling (Fig. 3).

Finally, within a bio-based approach, the selection of enzyme-biodegradation vs. whole-cell bioconversion is still an open question. For applications in which upcycling into value-added chemicals is key, whole-cell bioconversion will certainly be desirable. For in situ bioremediation-type applications, microbial processes that do not require any polymer pre-processing will be desired. For applications of recircularization, pure enzyme systems may be more advantageous and lead to easier downstream recovery without potential catabolism of breakdown products. Since isolated enzymes can work outside of the temperature, pH, and media conditions required for cell growth, it is possible to operate these pure enzyme systems at higher temperature or even in the presence of organic solvents or ionic liquids, which could bypass bottlenecks related to degradation efficiency or plastic properties such as high crystallinity^19^.

## Intrinsic barriers to efficient plastic biodegradation

**Fig. 4:**
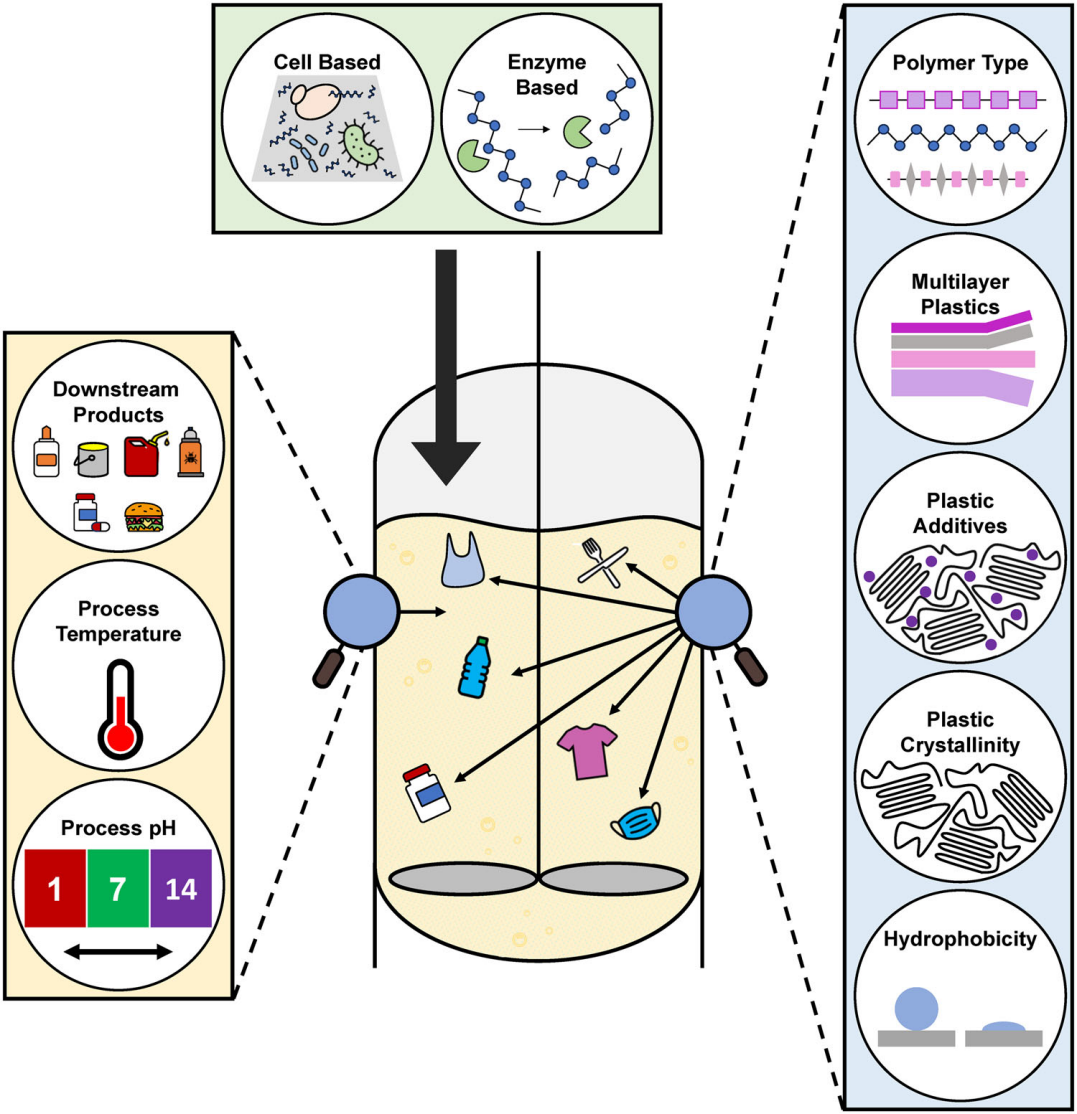
Bottlenecks to bio-based plastic degradation. Biological depolymerization of plastic can utilize whole-cell biocatalysts, purified enzymes or a combination of the two. Regardless of this choice, many of the remaining bottlenecks are rooted in the plastic’s material characteristics such as chemical composition/bond types, multilayered nature, additives, crystallinity, and hydrophobicity. Process parameters such as temperature, pH, and downstream products are dependent on the choice of biological approach but can also be leveraged to overcome various material trait bottlenecks.

Despite the possible advantages and distinct differences that biological depolymerization could offer, there are still significant intrinsic barriers that must be overcome using approaches such as enzyme engineering, strain discovery/evolution, and process engineering, and process optimization (Fig. 4). Some of these bottlenecks include high crystallinity, the inclusion of additives, and the often mixed/layered composition of plastics. The challenge of polymer crystallinity is a considerable roadblock for biological recycling and has led to many attempts at developing preprocessing methods^20–24^. However, while these preprocessing methods increase enzymatic degradation efficiency, many are not commercially/economically scalable. Outside of preprocessing to lower crystallinity, operations at temperatures at or above the glass transition temperature can reduce the impact of crystallinity^11^, however this may inadvertently contribute to plastic aging which can have the unintended effect of actually increasing crystallinity^25,26^. While recent efforts have successfully used machine learning and protein engineering to increase the thermostability of degradation enzymes, this approach only works for a small handful of plastics that have biologically-compatible glass transition temperatures. Further complicating this issue is the hydrophobic nature of most plastic surfaces—a desirable trait in the functional lifecycle of plastics, but detriment in biodepolymerization of these materials. Approaches that increase the adsorption of enzymes and microbes to these surfaces have the potential to increase biodegradation efficiency^27^. Likewise, applying enzyme technologies in non-conventional, non-aqueous environments including approaches of dry/moist-solid reactions and solvent-conditions are areas for further exploration^28^. Additional approaches to embed plastics with degrading enzymes are also being researched as an alternative method to allow for surface-enzyme interactions^29^. Notably, this method has seen success in the degradation of polyesters such as poly(lactic acid) and poly(caprolactone) resulting in substantial depolymerization into small molecules. However, this approach has proven to be more difficult when applied to polyolefins^30^.

In contrast to the relatively pure and pristine plastic used in most published papers, the reality of large-scale plastic circularization for a true biorefinery is the presence of not only virgin plastic, but recycled plastics as well as additives, plasticizers, non-plastic components (including inks, adhesives, and papers) and contaminations from food, biological, and chemical materials. These factors may have synergetic or antagonistic effects and as a result need to be studied for the role that they play in plastic degradation^31,32^. As a final layer of complexity, most input streams will not be pure and will certainly contain mixed plastics (either due to inefficiency of sorting or, more so, the abundant use of multi-layer plastics). This combination of factors makes it difficult to identify singular microbes or enzymes that can meet these comprehensive degradation needs. As a result, upcoming instantiations of circular plastic economies may evaluate whether a cocktail of enzymes or microbes behave better than singular enzyme/microbe selections for overall conversion rates and yields.

## Considerations and approaches to achieving biotechnological depolymerization

Plastic is an anthropogenic addition to the environment and thus most identified enzyme classes that act on these products derive from native enzyme promiscuity. Using “-omics” tools and machine learning, many plastic degrading enzymes and classes of organism have been discovered with the promise of further improved/unique activity yet to be discovered^33^. At present, multiple microbes and even isolated enzymes from these hosts have been identified that have some capacity to degrade plastic, discussed in more detail below for multiple types of polymers.

As described above, the choice of enzyme vs microbial process has strong considerations in the field. The most basic microbial depolymerization and conversion process is typically characterized by secretion of extracellular enzymes by the microbes, colonization of the plastic surface, hydrolysis of plastics to smaller molecules, and finally the assimilation/conversion of these smaller molecules into a product of interest. Unlike sugars from cellulose, the PET monomer terephthalic acid (TPA) has a lower degree of reduction than glucose. These differences indicate the importance of parity matching the chemical and redox potential of these depolymerized components with a desired valorized or upcycled product when using whole-cell biocatalysis. Upcycling the depolymerized components from plastics could be especially useful as a feedstock for industries that have market demands comparable to plastic production—such as food and fuel. In this regard, the upcycling of depolymerized plastic into only niche market molecules does not fully solve the challenge of a plastic circularization bioeconomy. While these approaches may demonstrate a potential, they do not represent a comprehensive solution.

## Commonly utilized plastics and their current biodegradation status

Given the prolific nature of plastic use, a number of naturally occurring and engineering solutions are beginning to emerge for biological depolymerization approaches. Here, we highlight the potential and state-of-the-art for many of the common plastics.

## Biodegradable plastics

A number of synthesized (some even bio-derived) plastics are already theoretically fully biodegradable through methods such as composting including products such as polylactide (PLA), polybutylene adipate terephthalate (PBAT), and polyhydroxybutyrate (PHB). Degrading these plastics, however, rely on microorganisms at very specific environmental conditions to achieve full depolymerization^34^. In this regard, these conditions can be rather challenging to achieve with typical at-home composting and, in some instances, even for industrial composting^35^. Of more concern, utilizing composting as an end-of-life option for these plastics promotes a more linear life cycle as they treat these plastics as single use. As a result, a continued supply of resources is required to re-synthesize these polymers. For fossil fuel-derived biodegradable plastics such as PBAT, composting methods deal with the individual plastic end-of-life, but may prove to be an environmental strain. Additionally, biodegradability may not be fully achievable in “non-ideal” conditions, thus leading to longer-than-expected persistence in the environment, especially in the form of microplastics. In contrast to the linear life cycle, enzymatic degradation and monomer recovery may offer the opportunity to establish a more circular life cycle. PLA for example can be degraded into its constituent monomer, lactic acid through the use of proteases^36^. Likewise, cutinases from *T. fusca* (TfCut) were shown to degrade PBAT into TPA and other monomer derivatives of PBAT^37^. While not conducted in large scale yet, these approaches may enable a circular loop for PLA and PBAT synthesis.

## Polyethylene terephthalate (PET)

Many organisms have been recently discovered with the capacity to degrading PET. The majority of these organisms are either bacterial or fungal and have been sampled from a wide variety of environments including landfills, soil, and water^38^. While some of these microorganisms can use PET as a sole carbon source, they do not consume PET at a rate necessary for industrial applications. Moreover, genetic tools are significantly lacking in many of these hosts organisms as exemplified by *Ideonalla sakaiensis* which only recently has been explored for gene knockout applications^39^. For more genetically tractable microbes, the utilization of PET (or in many cases, TPA) has been explored as a means to upcycle into other products such as PHB^40^.

While whole-cell PET degradation technologies are being developed, considerable progress and process realization have been made using purified enzymes from identified microorganisms to depolymerize PET into its constituent monomers: TPA, ethylene glycol (EG), and/or mono-(2-hydroxyethyl)terephthalic acid (MHET) (Fig. 3). The majority of these enzymes fall into the α/β-hydrolase fold enzyme superfamily^12,41^ and within that superfamily include many types of enzymes such as cutinases, esterases, and lipases^12,38^. Although wild-type enzymes such as TfH^42^, LCC^43^, *Is*PETase^44^, BhrPETase^45^, and PHL7^33^ can degrade PET in vitro, they generally have low activity on PET. In order to increase activity and thermostability, various enzyme engineering approaches have been utilized: rational engineering was used to generate the LCC quadruple mutant LCC^ICCG 11^ and the thermostable PETase variant ThermoPETase^46^, another thermostable PETase (HotPETase) variant was generated using directed evolution^47^, mutagenesis approaches were used to develop the PETase variant PES-H1 L92F/Q94Y for improved catalytic ability^48^, computational strategies were used to develop DuraPETase^49^, and a machine learning algorithm was used to develop FAST-PETase^22^. The tools developed and verified for the engineering of PET hydrolases will not only continue to improve but will certainly expedite catalytic improvements in the future for additional plastic-degrading enzymes. These engineered enzymes can be used in the microorganisms described above to improve whole-cell conversion of PET into upcycled products.

Enzymatic depolymerization of PET on its own allows for the subsequent reuse and re-polymerization of resulting monomers. These technologies have already migrated from bench and laboratory scale to industrial scale processes, though they are still hindered by low enzymatic activity (including to degrade the MHET into TPA and EG), costly pretreatments needed to enable degradation of highly crystalline PET starting materials, and limited activity at lower pH levels (which impact cost of downstream separations as well as bioprocess cost due to high base addition during the overall biodegradation process). While some work has already been done in this area^50^, further effort is necessary to optimize reactor conditions and enzyme loading. Likewise, improved enzyme activity and the removal of the pretreatment steps will be necessary to enable cost-effective enzymatic depolymerization on an even larger commercial scale. These limitations notwithstanding, the implementation of this technology at-scale by Carbios is a reminder of how enzymatic-based depolymerization of PET is an industrially-deployable technology, and not just an academic endeavor. Currently there are no industrial or commercial PET bio-recycling processes that utilize microorganisms, however this could be an effective avenue for PET recycling once developed, especially for in situ remediation aspects.

## Polyolefins (PO)

For efficient degradation of polyolefins such as PE and PP, it is likely that initial microorganisms and/or enzymes will be required to modify the hydrocarbon backbone thus making it more susceptible to enzyme cleavage. A handful of microbes with proposed PO degrading capabilities have already been identified^51^. In a way, this enzymatic capacity will be a biochemical preprocessing step. Recently reported enzymes with the ability to modify the hydrocarbon backbone include laccases, peroxidases, and hydroxylases^9,52–55^. A key feature of this modification process is the ability of the microbes to attack C–C bonds. This alteration of the C-C bonds is hypothesized to proceed in a manner analogous to the process of catabolizing long-chain alkanes which may include the hydroxylation of the C–C bonds resulting in alcohols^56^. This initial hydroxylation may be accompanied by further oxidation^9,57^. Oxygenation changes the resulting polymer traits and deviates from a pure polymer-to-polymer circularization scheme. These smaller chain by-products such as short chain fatty acids, ketones, aldehydes, and alkanes can then theoretically be used as upcycling feedstocks for other processes (biological or chemical) to produce valorized or upcycled products (Fig. 3). On a laboratory scale, PE already been upcycled to polyhydroxyalkanoates^58^, small molecules such as asperbenzaldehyde and citreoviridin^59^, and proteins such as spider silk^16^. While these products illustrate the possibility for upcycled products, processes need to be developed to parity match the volume of PE waste with downstream products at the same scale such as fuels and foods. However, optimal use of these feedstocks within a biorefinery scheme is important, as depolymerization products such as alkanes will likely have higher degrees of reduction compared with conventional feedstocks like glucose. While research into the biodegradation of POs is quickly advancing, additional work is needed before biodegradation can become an effective solution of PO plastic waste.

## Polyurethanes (PUR)

PURs are a wide and varied class of polymers, known for their durability, which incorporate urethane bonds in addition to polyester, polyether, and urea bonds. These plastics can processed either as a thermoplastic or thermoset depending on the formulation and production^60^. Due to the variation between PURs unlike a plastic like PET, there is no ideal or model substrate for research purposes and thus the substrate of choice has varied between studies. To date, many fungal and bacterial species have been identified to utilize PUR as a carbon source including many strains of *Pseudomonas* and *Aspergillus*^61,62^. The underlying enzymes responsible for PUR degradation have included urethanases^63^, esterases, lipases, cutinases, proteases, amidases, ureases, and oxidases (Fig. 3)^12,60–62^. This wide range of enzymatic function is not surprising given that PURs contain several types of bonds and functional groups. As such, the diversity within PURs creates difficulties in creating a PUR biorecycling system using a singular enzyme or microbe. However, these traits allow for many possible biorecycling schemes, such as selective degradation of a single subclass of PURs within a mixture or the use of microbial consortia to enable broad PUR degrading capabilities. Given the diverse set of monomers, it is likely that a biodegradation scheme for PURs will embody upcycling approaches.

## Other plastics: polyamides (PA), polystyrene (PS), and polyvinyl chloride (PVC)

While PET, PE, PP, and PUR account for four of the six most produced plastics, the third most produced plastic, PVC, and the sixth most produced^4^, PS, have limited research into their biodegradation potential^12^. Moreover, while PS is the sixth most produced plastic, it accounts for 30% of plastic found in landfills^64^. Many of the biodegradation efforts for PS have explored the use of insects (and the commensal microorganisms found within) as a means of both degradation and bioprospecting. In this regard, many insects and microbes known to degrade PS have limited activity taking several weeks to degrade small weight percent of material^65,66^. For example, the larvae of the *Tenebrio molitor* moth has been identified to degrade PS^67^ and PVC^68^ when fed a plastic only diet. From the gut microbiome of the *T. molitor*, candidate microorganisms for PS degradation have been identified^69^. Similarly, a bacterial consortia from *T. molitor* was shown to degrade additive free PVC (thus bypassing challenges in the literature wherein biodegradation of additives present in PVC are mistaken for degradation of the plastic)^70^. Further examples of PVC degrading microbes have been isolated the microbiomes of various larvae^71,72^ as well as from environmental samples^73,74^. The discovery of these insects and subsequent microbes is a first step in creating a biobased upcycling approach for PS and PVC. However, it must be emphasized that researchers should be cautious when interpreting the results from the early stages of this research, especially when there is little definitive molecular data on the degradation products to conclusively show total depolymerization and not solely fragmentation.

While not in the top six most produced plastics, polyamides (PAs) are a common synthetic fiber of importance, especially in the textile industry. PAs are also of interest as polymer precursors that can be produced microbially^75^ which opens the door to a microbe enabled circular economy bypassing the need for a fossil fuel derived starting product. There are various types of PAs, but the two major versions are nylon-6, and nylon-66. Microbes such as *Flavobacterium* sp. KI72 and *Pseudomonas* sp. NK87 are able to utilize nylon oligomers as a carbon source, but suffer from a low substrate utilization rate^76^. A variety of specific, isolated enzymes have been identified with some capability to degrade PAs including proteases, cutinases, and amidases^12^. From these enzyme classes, the amidase enzymes NylA, NylB, NylC, NylD, and NylE in particular have been studied for their ability to degrade oligomers of nylon-6 into its monomeric subunits^77^. With both whole cell and enzymatic opportunities for the biodegradation of PAs, there is the possibility to transform recycled textiles back into new textiles or to upcycle them into various other products. The methods for PA biodegradation are currently limited by low activity, however the fast-growing bank of knowledge about engineering enzymes and microbes for other plastics can be utilized to find solutions to this problem. One example is the cascade of enzymes currently being explored for the depolymerization of another fossil fuel-derived plastic, polyvinyl alcohols (PVA)^78^.

## Technoeconomic analysis can guide the future of biorecycling approaches

The challenges and opportunities of enzyme and microbial-based polymer depolymerization are best viewed in light of the entire system. In this regard, the decisions of both whether and how to utilize biology as a means establishing a polymer circular economy can be guided by a thorough technoeconomic assessment (TEAs) and lifecycle assessment (LCA). With many of these technologies still in their infancy, such analysis is rather limited. However, a recent TEA for enzyme catalyzed PET recycling underscores the impact of feedstock pricing and especially pretreatment as major contributors to the price of recycled PET^79^. Additionally, the extent of reaction, solids and enzyme loading, and enzyme cost also impact the minimum selling price for a regenerated product^79^. Operations at higher temperatures or the use organic solvents or ionic liquids could have benefits in reducing crystallinity and thus reduce the overall need for pretreatment. However, these approaches also contribute to higher start up and operational costs, especially for the recovery of these solvents which can involve difficult, energy-intensive separations. Higher solids loading or reduced enzyme purity would decrease the enzyme cost. In total, all of these factors can impact the feasibility of a process and thus the TEA results.

Similarly, LCA of enzymatic PET recycling also highlights mechanical pretreatment alongside the use of sodium hydroxide and energy usage as major detriments in the impact categories of human health, natural resources, and natural environment^80^. Thus, finding methods for depolymerization which do not necessitate highly buffered reaction solutions or identifying more environmentally friendly chemicals would lessen the impact of current enzymatic depolymerization technologies. In terms of energy usage, relying on renewable energy sources instead of current conventional energy would certainly positively impact LCA results.

These examples highlight how using TEAs and LCAs can and should guide researchers in their efforts to develop better technologies for plastic depolymerization. TEA and LCA not only point toward specific areas for the improvement of enzymatic plastic depolymerization but also highlight that biorecycling is not viable as the sole solution to the plastic waste crisis. In this regard, bioprocessing plastic waste is a complementary technique to mechanical and chemical recycling alongside a reduction in use by individuals and industry.

## Final remarks

Great advances have been made in both identifying and engineering enzymes and microbes for the biological depolymerization of plastic. Certainly, further application of synthetic biology and directed evolution will aid researchers towards engineering better microbes and enzymes for plastic degradation. Coupling technology with economic incentives, financial and policy support, and waste infrastructure alterations will be necessary to fully shift biobased approaches to plastic degradation from the lab bench into a full-scale industrial process that helps to solve our plastic waste problem. The few technologies that are being used in an industrial setting are still in their first stages and not yet deployed at the scale and ease (i.e., without the need for any preprocessing) needed to compete/displace mechanically recycled, let alone, virgin plastic material. For any significant progress to be made in tackling the plastic waste problem, a concerted global effort is needed. These efforts may come in the form of the implementation of environmental policies both on the national and global scales. Policies like these prompt companies to adopt a more sustainability focused approach as was seen with the establishment of the United Nations’ sustainable development goals^81^. Looking at plastic waste degradation through a sustainability lens, such environmental policies may prompt companies to explore more environmentally benign methods to tackle plastic degradation such as the aforementioned biobased depolymerization schemes. On the environmental side, in situ remediation would most likely not allow for the use of the carbon from the plastic in a circular manner for reuse, but would be an important part of plastic waste management. Taken together, plastic can serve as a potent feedstock for both bio-enabled recycling and upcycling. The power of biology to depolymerize this material is vast and just beginning to be explored. With further efforts, we believe that these challenges can become debottlenecked and the true vision of a circular plastics bioeconomy could be realized.
